# 1-[1-(2,1,3-Benzoxadiazol-5-ylmeth­yl)-1*H*-1,2,3-triazol-4-yl]hexan-1-one

**DOI:** 10.1107/S1600536812041839

**Published:** 2012-10-13

**Authors:** Jessie A. Key, Christopher W. Cairo, Robert McDonald

**Affiliations:** a Alberta Glycomics Centre, Department of Chemistry, University of Alberta, Edmonton, AB T6G 2G2, Canada; b X-ray Crystallography Laboratory, Department of Chemistry, University of Alberta, Edmonton, AB T6G 2G2, Canada

## Abstract

The title compound, C_15_H_17_N_5_O_2_, was synthesized as part of a series of benzoxadiazole analogs which were examined for fluorescent properties by Cu-catalysed azide–alkyne cyclo­addition (CuAAC) of a 4-azido­methyl-benzoxadiazole substrate. The structure shows a nearly coplanar orientation of the hexa­none keto group and the 1,2,3-triazole ring [dihedral angle = 4.3 (3)°], while the benzoxadiazole and triazole groups are much more severely inclined [dihedral angle = 70.87 (4)°]. In the crystal, weak C—H⋯N inter­actions connect translationally-related triazole rings, while another set of C—H⋯N inter­actions is formed between inversion-related benzoxadiazole units, forming a three-dimensional network. The crystal studied was a non-merohedral twin with refined value of the twin fraction of 0.2289 (16).

## Related literature

For the synthesis of similar benzoxadiazole compounds, see: Key & Cairo (2011 ▶); Li *et al.* (2010 ▶). For two related benzoxadiazole-triazole structures, see: Key, Cairo & Ferguson (2012 ▶); Key, Cairo & McDonald (2012 ▶). For structures of 1-(ar­yl)methyl-1,2,3-triazole compounds with 4-carbonyl substituents [*R*C(O) or *R*OC(O)], see: Harju *et al.* (2003 ▶); Huang *et al.* (2010 ▶); Dong & Cheng (2011 ▶); Jia & Lu (2011 ▶); Menendez *et al.* (2012 ▶). 
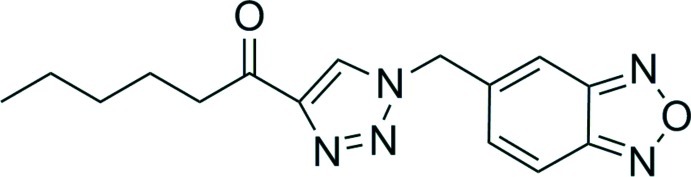


## Experimental

### 

#### Crystal data

C_15_H_17_N_5_O_2_
*M* *_r_* = 299.34
Monoclinic, 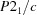

*a* = 16.5752 (16) Å
*b* = 5.5429 (5) Å
*c* = 16.2452 (16) Å
β = 91.3612 (13)°
*V* = 1492.1 (2) Å^3^
*Z* = 4
Mo *K*α radiation
μ = 0.09 mm^−1^
*T* = 173 K
0.74 × 0.14 × 0.06 mm

#### Data collection

Bruker APEXII CCD diffractometer
Absorption correction: multi-scan (TWINABS; Bruker, 2008 ▶) *T*_min_ = 0.935, *T*_max_ = 0.995
45733 measured reflections
3079 independent reflections
2413 reflections with *I* > 2σ(*I*)
*R*_int_ = 0.051

#### Refinement

*R*[*F* ^2^ > 2σ(*F* ^2^)] = 0.040
*wR*(*F* ^2^) = 0.091
*S* = 1.03
3079 reflections
201 parameters
H-atom parameters constrained
Δρ_max_ = 0.19 e Å^−3^
Δρ_min_ = −0.17 e Å^−3^

### 

Data collection: *APEX2* (Bruker, 2008 ▶); cell refinement: *SAINT* (Bruker, 2008 ▶); data reduction: *SAINT*; program(s) used to solve structure: *SHELXD* (Sheldrick, 2008 ▶); program(s) used to refine structure: *SHELXL97* (Sheldrick, 2008 ▶); molecular graphics: *SHELXTL* (Sheldrick, 2008 ▶); software used to prepare material for publication: *publCIF* (Westrip, 2010 ▶).

## Supplementary Material

Crystal structure: contains datablock(s) I, New_Global_Publ_Block. DOI: 10.1107/S1600536812041839/mw2084sup1.cif

Structure factors: contains datablock(s) I. DOI: 10.1107/S1600536812041839/mw2084Isup2.hkl

Supplementary material file. DOI: 10.1107/S1600536812041839/mw2084Isup3.cml

Additional supplementary materials:  crystallographic information; 3D view; checkCIF report

## Figures and Tables

**Table 1:** Hydrogen-bond geometry (Å, °)

*D*—H⋯*A*	*D*—H	H⋯*A*	*D*⋯*A*	*D*—H⋯*A*
C3—H3⋯N2^i^	0.95	2.58	3.475 (2)	158
C8—H8⋯N5^ii^	0.95	2.41	3.350 (2)	171

Symmetry codes: (i) 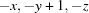; (ii) 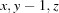.

**Fig. 1.**
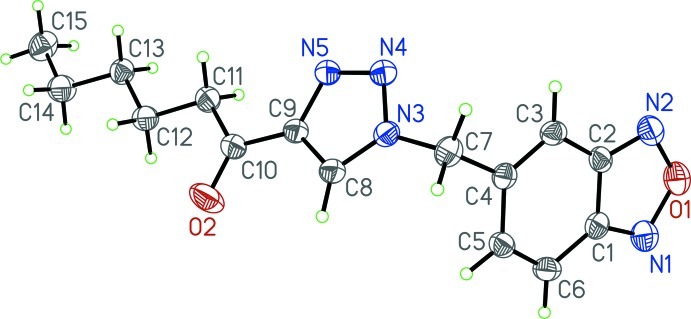
View of I. Non-hydrogen atoms are represented by Gaussian ellipsoids at the 50% probability level. Hydrogen atoms are represented with artificially small thermal parameters.

**Fig. 2.**
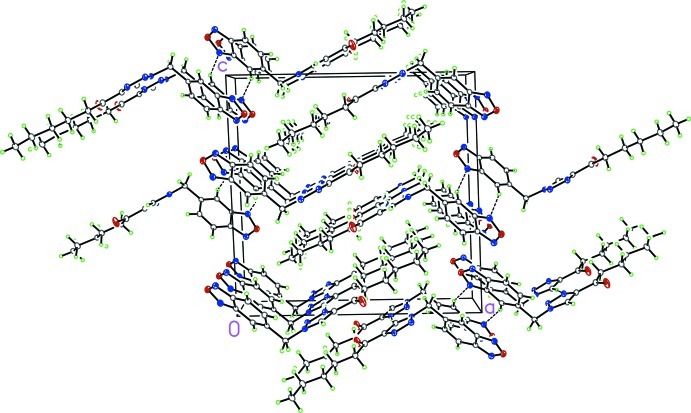
Illustration of crystal packing as viewed parallel to the crystal b axis. Nonbonded C-H···N interactions are shown with dashed lines (see Table 1).

**Fig. 3.**
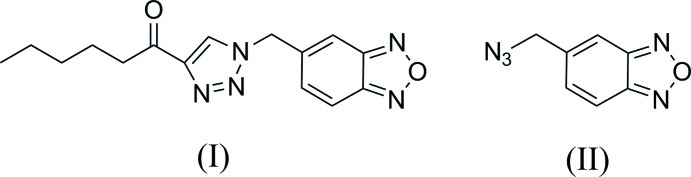
Compounds used in this study.

## supplementary crystallographic information

### Comment

In an effort to explore benzoxadiazole derivatives with interesting spectroscopic properties, we generated the title compound, I, for comparison to its parent 4-azidomethyl-benzoxadiazole (II). Although we observed large changes in the spectra of substrates with an azido group conjugated to the chromophore, derivatives with an intervening methylene group tended to have only small changes upon triazole formation (Key & Cairo, 2011). Compound I was synthesized unintentionally through the use of an oxidized sample of *n*-octyne which also contained oct-1-yn-3-one as an impurity. The hexanone product was isolated by column chromatography and used for structural studies. (λ_max_*_ABS_* EtOH: 278, 289 nm, Absorption coefficient: 12,300 *M*^-1^cm^-1^)

In the crystal the 1,2,3-triazole ring and hexan-1-on-1-yl groups are nearly coplanar; the angle between the ketonic fragment (O2-C10-C11) and the 1,2,3-triazole ring is 4.3 (3)°. The rings of the benzoxadiazole and triazole groups are much more severely inclined (70.87 (4)°). Weak C-H···N interactions are observed between triazole moieties related via translation parallel to the *b* axis (2.41 Å for H8···N5[*x*, -1+*y*, *z*]). A further set of weak C-H···N interactions is seen between benzoxadiazole groups related by the inversion center (0, ^1^/_2_, 0) (2.58 Å for H3···N2[-*x*, 1-*y*, -*z*]). A parallel-stacking interaction is observed between benzoxadiazole rings related by inversion through the origin (interplanar spacing = 3.328 Å).

### Experimental

4-(Azidomethyl)benz-[2,1,3-*d*]-oxadiazole (II) (40 mg, 0.23 mmol, 1 equiv) was dissolved in 1:1 water/methanol (5 mL). To this solution was added *n*-octyne (0.17 mL, 1.14 mmol, 5 equiv), which also contained oct-1-yn-3-one as an impurity. Copper sulfate (7 mg, 0.046 mmol, 0.2 equiv) and ascorbic acid (12 mg, 0.068 mmol, 0.3 equiv) were then added to the solution. The reaction mixture was allowed to stir at room temperature for approximately 1 h, forming a red precipitate. The precipitate was filtered off and the product was obtained after purification by column chromatography (EtOAc/hexanes) (35 mg, 51% yield). ^1^H NMR (400 MHz, CDCl_3_): δ 8.16 (s, 1H), 7.89 (d, 1H, ^3^*J* = 9.2 Hz), 7.51 (s, 1H), 7.32 (dd, 1H, ^4^*J* = 1.2 Hz, ^3^*J* = 9.2 Hz), 5.70 (s, 2H), 3.11(t, 2H, ^3^*J* = 7.2 Hz), 1.74 (m, 2H), 1.36 (m, 4H), 0.89 (m, 3H); ^13^C NMR (100 MHz, CDCl_3_): δ 195.5, 149.1, 148.9, 148.8, 138.1, 131.1, 126.0, 118.5, 116.0, 54.1, 39.8, 31.6, 23.8, 22.7, 14.2; IR (microscope): ν = 3098, 2954, 2930, 1691, 1531 cm^-1^; ES-HRMS calculated for C_15_H_17_N_5_O_2_Na [*M*+Na]^+^: 322.1279; observed: 322.1279. *R*_f_ = 0.45 (1:1 EtOAc/hexanes).

### Refinement

The crystal used for data collection was found to display non-merohedral twinning. Both components of the twin were indexed with the program *CELL_NOW* (Bruker, 2008). The second twin component can be related to the first component by 180° rotation about the [-1 0 2] axis in both real and reciprocal space. A raw data file was produced by the data integration program *SAINT* (Bruker, 2008), using the two-component orientation matrix file that had been produced by *CELL_NOW*. Integrated intensities for the reflections from the two components were written into a *SHELXL-97* HKLF 5 reflection file with the program *TWINABS* (Bruker, 2008), using all reflection data (exactly overlapped, partially overlapped and non-overlapped). The reflection (1 0 0) was found to have an excessively high disagreement between *F*_o_ and *F*_c_, and was omitted from the refinement. The refined value of the twin fraction (*SHELXL-97* BASF parameter) was 0.2289 (16). All H atoms were generated in idealized positions and refined using a riding model with fixed C-H distances (C-H_aromatic_ = 0.95 Å, C-H_methylene_ = 0.99 Å, C-H_methyl_ = 0.98 Å) and with *U*_iso_(H) = 1.2*U*_eq_(C).

### Crystal data

**Table:**

C_15_H_17_N_5_O_2_	*F*(000) = 632
*M**_r_* = 299.34	*D*_x_ = 1.333 Mg m^−^^3^
Monoclinic, *P*2_1_/*c*	Mo *K*α radiation, λ = 0.71073 Å
Hall symbol: -P 2ybc	Cell parameters from 5974 reflections
*a* = 16.5752 (16) Å	θ = 2.5–24.3°
*b* = 5.5429 (5) Å	µ = 0.09 mm^−^^1^
*c* = 16.2452 (16) Å	*T* = 173 K
β = 91.3612 (13)°	Rod, colourless
*V* = 1492.1 (2) Å^3^	0.74 × 0.14 × 0.06 mm
*Z* = 4	

### Data collection

**Table:**

Bruker APEXII CCD diffractometer	3079 independent reflections
Radiation source: fine-focus sealed tube	2413 reflections with *I* > 2σ(*I*)
Graphite monochromator	*R*_int_ = 0.051
Detector resolution: 8.26 pixels mm^-1^	θ_max_ = 26.5°, θ_min_ = 1.2°
ω scans	*h* = −20→20
Absorption correction: multi-scan (TWINABS; Bruker, 2008)	*k* = −6→6
*T*_min_ = 0.935, *T*_max_ = 0.995	*l* = −20→20
45733 measured reflections	

### Refinement

**Table:**

Refinement on *F*^2^	Secondary atom site location: difference Fourier map
Least-squares matrix: full	Hydrogen site location: inferred from neighbouring sites
*R*[*F*^2^ > 2σ(*F*^2^)] = 0.040	H-atom parameters constrained
*wR*(*F*^2^) = 0.091	*w* = 1/[σ^2^(*F*_o_^2^) + (0.0331*P*)^2^ + 0.4646*P*] where *P* = (*F*_o_^2^ + 2*F*_c_^2^)/3
*S* = 1.03	(Δ/σ)_max_ < 0.001
3079 reflections	Δρ_max_ = 0.19 e Å^−^^3^
201 parameters	Δρ_min_ = −0.17 e Å^−^^3^
0 restraints	Extinction correction: *SHELXL*, Fc^*^=kFc[1+0.001xFc^2^λ^3^/sin(2θ)]^-1/4^
Primary atom site location: structure-invariant direct methods	Extinction coefficient: 0.0114 (15)

### Special details

**Table:**

Geometry. All standard uncertainties (s.u.'s) (except the s.u. in the dihedral angle between two least-squares planes) are estimated using the full covariance matrix. The cell s.u.'s are taken into account individually in the estimation of s.u.'s in distances, angles and torsion angles; correlations between s.u.'s in cell parameters are only used when they are defined by crystal symmetry. An approximate (isotropic) treatment of cell s.u.'s is used for estimating s.u.'s involving least-squares planes.
Refinement. Refinement of *F*^2^ against ALL reflections. The weighted *R*-factor *wR* and goodness of fit *S* are based on *F*^2^, conventional *R*-factors *R* are based on *F*, with *F* set to zero for negative *F*^2^. The threshold expression of *F*^2^ > σ(*F*^2^) is used only for calculating *R*-factors(gt) *etc*. and is not relevant to the choice of reflections for refinement. *R*-factors based on *F*^2^ are statistically about twice as large as those based on *F*, and *R*- factors based on ALL data will be even larger.

### Fractional atomic coordinates and isotropic or equivalent isotropic displacement parameters (Å^2^)

**Table:**

	*x*	*y*	*z*	*U*_iso_*/*U*_eq_	
O1	−0.07380 (7)	0.1919 (2)	0.16041 (7)	0.0409 (3)	
O2	0.51268 (8)	0.0088 (2)	0.11538 (10)	0.0593 (4)	
N1	−0.03444 (8)	−0.0165 (3)	0.18558 (9)	0.0382 (4)	
N2	−0.03404 (8)	0.3110 (3)	0.09850 (9)	0.0363 (3)	
N3	0.29027 (7)	0.1559 (2)	0.00229 (8)	0.0272 (3)	
N4	0.29675 (8)	0.3994 (2)	0.00904 (9)	0.0317 (3)	
N5	0.36597 (7)	0.4428 (2)	0.04758 (9)	0.0309 (3)	
C1	0.02958 (9)	−0.0270 (3)	0.13892 (9)	0.0287 (4)	
C2	0.02964 (8)	0.1752 (3)	0.08476 (9)	0.0272 (3)	
C3	0.09143 (9)	0.2061 (3)	0.02610 (9)	0.0278 (3)	
H3	0.0918	0.3406	−0.0101	0.033*	
C4	0.14968 (9)	0.0339 (3)	0.02451 (9)	0.0256 (3)	
C5	0.14964 (9)	−0.1700 (3)	0.07982 (10)	0.0289 (3)	
H5	0.1919	−0.2850	0.0766	0.035*	
C6	0.09180 (9)	−0.2039 (3)	0.13617 (10)	0.0311 (4)	
H6	0.0927	−0.3392	0.1721	0.037*	
C7	0.21712 (9)	0.0526 (3)	−0.03636 (10)	0.0314 (4)	
H7A	0.2294	−0.1098	−0.0580	0.038*	
H7B	0.1994	0.1553	−0.0833	0.038*	
C8	0.35461 (9)	0.0453 (3)	0.03618 (10)	0.0293 (4)	
H8	0.3646	−0.1233	0.0395	0.035*	
C9	0.40295 (9)	0.2287 (3)	0.06506 (10)	0.0288 (4)	
C10	0.48268 (9)	0.2074 (3)	0.10742 (10)	0.0330 (4)	
C11	0.52284 (9)	0.4320 (3)	0.13876 (11)	0.0331 (4)	
H11A	0.4886	0.5057	0.1811	0.040*	
H11B	0.5268	0.5484	0.0928	0.040*	
C12	0.60693 (9)	0.3892 (3)	0.17585 (11)	0.0349 (4)	
H12A	0.6029	0.2767	0.2229	0.042*	
H12B	0.6409	0.3117	0.1341	0.042*	
C13	0.64772 (9)	0.6203 (3)	0.20530 (10)	0.0320 (4)	
H13A	0.6496	0.7355	0.1588	0.038*	
H13B	0.6149	0.6941	0.2488	0.038*	
C14	0.73289 (10)	0.5801 (3)	0.23904 (12)	0.0422 (4)	
H14A	0.7665	0.5154	0.1946	0.051*	
H14B	0.7315	0.4576	0.2833	0.051*	
C15	0.77195 (11)	0.8090 (4)	0.27312 (11)	0.0450 (5)	
H15A	0.8265	0.7723	0.2939	0.054*	
H15B	0.7397	0.8722	0.3180	0.054*	
H15C	0.7748	0.9300	0.2293	0.054*	

### Atomic displacement parameters (Å^2^)

**Table:**

	*U*^11^	*U*^22^	*U*^33^	*U*^12^	*U*^13^	*U*^23^
O1	0.0309 (6)	0.0481 (8)	0.0440 (7)	0.0059 (6)	0.0048 (5)	−0.0042 (6)
O2	0.0466 (8)	0.0264 (7)	0.1034 (13)	0.0055 (6)	−0.0278 (8)	−0.0033 (7)
N1	0.0341 (7)	0.0428 (9)	0.0379 (8)	0.0016 (7)	0.0028 (6)	−0.0012 (7)
N2	0.0316 (7)	0.0373 (8)	0.0400 (8)	0.0036 (6)	−0.0019 (6)	−0.0019 (7)
N3	0.0270 (6)	0.0249 (7)	0.0297 (7)	−0.0020 (5)	0.0035 (5)	−0.0010 (6)
N4	0.0301 (7)	0.0256 (7)	0.0395 (8)	−0.0010 (6)	0.0011 (6)	0.0004 (6)
N5	0.0277 (7)	0.0242 (7)	0.0409 (8)	−0.0001 (5)	0.0004 (6)	0.0006 (6)
C1	0.0264 (8)	0.0317 (9)	0.0279 (8)	−0.0042 (7)	−0.0016 (6)	−0.0018 (7)
C2	0.0244 (7)	0.0258 (8)	0.0310 (8)	−0.0003 (6)	−0.0061 (6)	−0.0037 (7)
C3	0.0304 (8)	0.0248 (8)	0.0281 (8)	−0.0035 (7)	−0.0041 (6)	0.0008 (7)
C4	0.0254 (7)	0.0259 (8)	0.0254 (8)	−0.0039 (6)	−0.0045 (6)	−0.0033 (6)
C5	0.0281 (8)	0.0250 (8)	0.0334 (8)	0.0024 (6)	−0.0024 (6)	−0.0016 (7)
C6	0.0354 (8)	0.0256 (8)	0.0323 (9)	−0.0004 (7)	−0.0019 (7)	0.0037 (7)
C7	0.0311 (8)	0.0339 (9)	0.0292 (9)	−0.0043 (7)	−0.0012 (6)	−0.0042 (7)
C8	0.0288 (8)	0.0235 (8)	0.0359 (9)	0.0006 (6)	0.0040 (6)	0.0007 (7)
C9	0.0277 (8)	0.0228 (8)	0.0359 (9)	0.0004 (6)	0.0036 (6)	−0.0001 (7)
C10	0.0290 (8)	0.0258 (9)	0.0442 (10)	0.0011 (7)	−0.0009 (7)	0.0014 (8)
C11	0.0287 (8)	0.0285 (9)	0.0422 (10)	−0.0003 (7)	−0.0007 (7)	−0.0011 (7)
C12	0.0323 (8)	0.0312 (9)	0.0410 (10)	−0.0020 (7)	−0.0037 (7)	0.0050 (8)
C13	0.0295 (8)	0.0344 (9)	0.0321 (9)	−0.0017 (7)	−0.0005 (7)	0.0001 (7)
C14	0.0370 (9)	0.0402 (11)	0.0488 (11)	−0.0043 (8)	−0.0115 (8)	0.0055 (9)
C15	0.0410 (10)	0.0518 (12)	0.0418 (11)	−0.0113 (9)	−0.0077 (8)	0.0025 (9)

### Geometric parameters (Å, º)

**Table:**

O1—N2	1.3831 (18)	C7—H7B	0.9900
O1—N1	1.3835 (18)	C8—C9	1.370 (2)
O2—C10	1.214 (2)	C8—H8	0.9500
N1—C1	1.320 (2)	C9—C10	1.480 (2)
N2—C2	1.320 (2)	C10—C11	1.495 (2)
N3—C8	1.3376 (19)	C11—C12	1.524 (2)
N3—N4	1.3581 (18)	C11—H11A	0.9900
N3—C7	1.4682 (19)	C11—H11B	0.9900
N4—N5	1.3160 (17)	C12—C13	1.520 (2)
N5—C9	1.3627 (19)	C12—H12A	0.9900
C1—C2	1.425 (2)	C12—H12B	0.9900
C1—C6	1.425 (2)	C13—C14	1.519 (2)
C2—C3	1.426 (2)	C13—H13A	0.9900
C3—C4	1.358 (2)	C13—H13B	0.9900
C3—H3	0.9500	C14—C15	1.523 (2)
C4—C5	1.444 (2)	C14—H14A	0.9900
C4—C7	1.513 (2)	C14—H14B	0.9900
C5—C6	1.354 (2)	C15—H15A	0.9800
C5—H5	0.9500	C15—H15B	0.9800
C6—H6	0.9500	C15—H15C	0.9800
C7—H7A	0.9900		
			
N2—O1—N1	112.57 (11)	N5—C9—C10	123.94 (14)
C1—N1—O1	104.30 (13)	C8—C9—C10	127.49 (14)
C2—N2—O1	104.33 (13)	O2—C10—C9	118.73 (15)
C8—N3—N4	111.20 (12)	O2—C10—C11	122.74 (14)
C8—N3—C7	129.75 (13)	C9—C10—C11	118.53 (14)
N4—N3—C7	119.02 (13)	C10—C11—C12	113.64 (14)
N5—N4—N3	106.65 (12)	C10—C11—H11A	108.8
N4—N5—C9	108.83 (12)	C12—C11—H11A	108.8
N1—C1—C2	109.40 (14)	C10—C11—H11B	108.8
N1—C1—C6	129.85 (15)	C12—C11—H11B	108.8
C2—C1—C6	120.73 (14)	H11A—C11—H11B	107.7
N2—C2—C1	109.39 (14)	C13—C12—C11	112.92 (14)
N2—C2—C3	129.38 (15)	C13—C12—H12A	109.0
C1—C2—C3	121.22 (14)	C11—C12—H12A	109.0
C4—C3—C2	116.78 (14)	C13—C12—H12B	109.0
C4—C3—H3	121.6	C11—C12—H12B	109.0
C2—C3—H3	121.6	H12A—C12—H12B	107.8
C3—C4—C5	121.82 (14)	C14—C13—C12	113.13 (14)
C3—C4—C7	120.05 (14)	C14—C13—H13A	109.0
C5—C4—C7	118.13 (14)	C12—C13—H13A	109.0
C6—C5—C4	122.70 (15)	C14—C13—H13B	109.0
C6—C5—H5	118.6	C12—C13—H13B	109.0
C4—C5—H5	118.6	H13A—C13—H13B	107.8
C5—C6—C1	116.74 (15)	C13—C14—C15	113.11 (15)
C5—C6—H6	121.6	C13—C14—H14A	109.0
C1—C6—H6	121.6	C15—C14—H14A	109.0
N3—C7—C4	111.25 (12)	C13—C14—H14B	109.0
N3—C7—H7A	109.4	C15—C14—H14B	109.0
C4—C7—H7A	109.4	H14A—C14—H14B	107.8
N3—C7—H7B	109.4	C14—C15—H15A	109.5
C4—C7—H7B	109.4	C14—C15—H15B	109.5
H7A—C7—H7B	108.0	H15A—C15—H15B	109.5
N3—C8—C9	104.76 (14)	C14—C15—H15C	109.5
N3—C8—H8	127.6	H15A—C15—H15C	109.5
C9—C8—H8	127.6	H15B—C15—H15C	109.5
N5—C9—C8	108.57 (13)		
			
N2—O1—N1—C1	−0.28 (17)	C2—C1—C6—C5	−0.3 (2)
N1—O1—N2—C2	0.55 (16)	C8—N3—C7—C4	94.95 (19)
C8—N3—N4—N5	0.04 (18)	N4—N3—C7—C4	−82.78 (18)
C7—N3—N4—N5	178.17 (12)	C3—C4—C7—N3	98.31 (16)
N3—N4—N5—C9	0.00 (17)	C5—C4—C7—N3	−81.66 (17)
O1—N1—C1—C2	−0.09 (16)	N4—N3—C8—C9	−0.05 (17)
O1—N1—C1—C6	−178.92 (15)	C7—N3—C8—C9	−177.93 (14)
O1—N2—C2—C1	−0.58 (16)	N4—N5—C9—C8	−0.03 (18)
O1—N2—C2—C3	178.43 (14)	N4—N5—C9—C10	179.26 (15)
N1—C1—C2—N2	0.45 (18)	N3—C8—C9—N5	0.05 (17)
C6—C1—C2—N2	179.40 (14)	N3—C8—C9—C10	−179.21 (15)
N1—C1—C2—C3	−178.66 (14)	N5—C9—C10—O2	−175.34 (17)
C6—C1—C2—C3	0.3 (2)	C8—C9—C10—O2	3.8 (3)
N2—C2—C3—C4	−178.81 (15)	N5—C9—C10—C11	4.8 (2)
C1—C2—C3—C4	0.1 (2)	C8—C9—C10—C11	−176.09 (16)
C2—C3—C4—C5	−0.4 (2)	O2—C10—C11—C12	4.1 (3)
C2—C3—C4—C7	179.59 (13)	C9—C10—C11—C12	−176.03 (15)
C3—C4—C5—C6	0.4 (2)	C10—C11—C12—C13	178.46 (14)
C7—C4—C5—C6	−179.61 (14)	C11—C12—C13—C14	−177.59 (15)
C4—C5—C6—C1	0.0 (2)	C12—C13—C14—C15	−176.65 (15)
N1—C1—C6—C5	178.39 (16)		

### Hydrogen-bond geometry (Å, º)

**Table:**

*D*—H···*A*	*D*—H	H···*A*	*D*···*A*	*D*—H···*A*
C3—H3···N2^i^	0.95	2.58	3.475 (2)	158
C8—H8···N5^ii^	0.95	2.41	3.350 (2)	171

Symmetry codes: (i) −*x*, −*y*+1, −*z*; (ii) *x*, *y*−1, *z*.
